# Quantum key distribution with prepare-and-measure Bell test

**DOI:** 10.1038/srep35032

**Published:** 2016-10-13

**Authors:** Yong-gang Tan

**Affiliations:** 1 Physics and Information Engineering Department, Luoyang Normal College, Luoyang 471022, Henan, People’s Republic of China

## Abstract

The prepare-and-measure quantum key distribution (QKD) has the merits of fast speed, high key generation rate, and easy implementation. However, the detector side channel attacks greatly undermine the security of the key bits. The eavesdropper, Eve, exploits the flaws of the detectors to obtain illegal information without violating quantum principles. It means that she can intervene in the communication without being detected. A prepare-and-measure Bell test protocol will be proposed. By randomly carrying out Bell test at the side of the information receiver, Bob, Eve’s illegal information gain within the detector side channel attack can be well bounded. This protocol does not require any improvement on the detectors used in available prepare-and-measure QKD. Though we only illustrate its application in the BB84 protocol, it is applicable for any prepare-and-measure QKD.

QKD is a real-time art of generating secure key bit string between remote partners^1^,^2^,^3^. Its security is not based on the computational complexity, but on the correctness of physical principles^4^,^5^,^6^,^7^. In practical conditions where imperfectly experimental devices are used, it is proven that secure key bit string can still be generated when the tagged key bits are well restricted^8^. For example, phase-randomized weaken coherent sources are used in practical QKD. There are multi-photon pulses emitted from the source. Eve can launch the photon-number-splitting (PNS) to tag the multi-photon events^9^,^10^. If the amount of the tagged event can be well bounded, with decoy state technology for example, secure key bits can be generated between remote partners^11^,^12^,^13^,^14^.

Recently, the detector side channel attacks have attracted great attention. Eve exploits the drawbacks of the detectors to control the detections. Moreover, the photons registered by the detectors may be not the ones expected by Bob, but well devised by Eve to obtained illegal information. In the fake state attack, Eve intercepts the photons from Alice and reads out the bit values on them. According to her measurement outcomes, she exploits the detection efficiency mismatches and prepares a fake state to be detected by Bob^15^. The time-shift attack uses the detection efficiency mismatches to eavesdrop on the communication without intercepting on Alice’s photons^16^,^17^. Furthermore, the problem of information leaking from the detector side channels also exists in the blinding attack^18^,^19^,^20^ and the phase re-mapping attack^21^,^22^.

The detector side channel attacks do great harm to the security of QKD because Eve’s illegal information gain obtained in the attacks cannot be well bounded^8^. Great improvement must be made on the detectors to avoid the detector side channel attacks^19^,^23^,^24^,^25^. It has been shown that alternative ways of measurements can be used to beat these attacks^26^,^27^,^28^,^29^,^30^,^31^,^32^. In this case, the security of the measurement outcomes relies on the monogamy of entanglement^33^,^34^. Accordingly, the experimental realization is more complex and the key generation rate is lower when compared with the prepare-and-measure QKD. An easy way to beat the detector side channel attack from the physics principle is expected.

Quantum theory is exclusive with the local hidden variable (lhv) theory^35^,^36^,^37^. Loophole-free Bell violation means that the lhv theory can be excluded. Or else, if no Bell violation can be obtained in the loophole-free Bell test, the quantum theory is incorrect. Recently, Bell violation is experimentally obtained with all loopholes are closed^38^,^39^,^40^. These significant results mean the lhvs do not exist. Based on this fact, the detector side channel attack in the prepare-and-measure QKD can be beat with a simple but efficient way. Random Bell test is required to be carried out at Bob’s side to check the quantum correlations between Alice and Bob. Though this protocol is devised for the BB84 protocol^1^, it is applicable to any prepare-and-measure QKD.

## The prepare-and-measure Bell test

In the Bell test, a parametric-down-conversion (PDC) source is set between Alice and Bob. Entangled photon pairs are generated and distributed to them. Alice has two sets of two-channel measurement devices, *A*_1_ and *A*_2_. Similarly, Bob has two sets of two-channel measurement settings, *B*_1_ and *B*_2_. Alice and Bob randomly choose their measurement settings to measure their incoming photons. The binarily possible measurement outcomes obtained from the measurement settings are assigned with −1 and 1. Alice and Bob use their basis choices and measurement outcomes to calculate the CHSH polynomial^37^


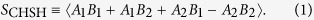


Here 〈*A*_*i*_〉, 〈*B*_*j*_〉 are the average values generated on *A*_*i*_ and *B*_*j*_, with *i*, *j* ∈ {1, 2}.

In local hidden variable theory and classical physics, the measurement outcomes at Alice’s side cannot be affected by those at Bob’s side. Similarly, the measurement outcomes at Bob’s side cannot be affected by those at Alice’s side, namely, the relation 〈*A*_*i*_*B*_*j*_〉 = 〈*A*_*i*_〉 〈*B*_*j*_〉 obeys^41^. Because −1 ≤ 〈*A*_*i*_〉 ≤ 1 and −1 ≤ 〈*B*_*j*_〉 ≤ 1 should be satisfied, *S*_CHSH_ varies from −2 to 2. Quantum-mechanically, its lower bound and upper bound are 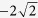 and 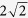, respectively^42^. Now that the Bell violation has been obtained with loophole-free Bell test^38^,^39^,^40^, the lhv theory does not need to be considered. If the experiment is not artificially controlled, Bell violation certifies the existence of entanglement.

Suppose that the entangled photon pair generated from the PDC source is encoded with 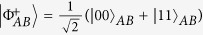, where the subscripts *A* and *B* denote Photon *A* and Photon *B*, respectively. After the photon pairs generated from the PDC source, Photon *A* is distributed to Alice, while Photon *B* is sent to Bob. In order to maximize the Bell violation, confinements are put on their basis choices. Without loss of any generality, one can assume that 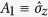, 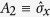, 
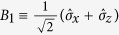
, and 
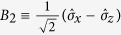
, where 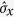 and 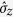 are the Pauli operators. Because the state 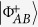 is rotationally invariant in the *X* − *Z* plane, one can obtain that 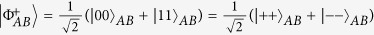, with 
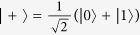
 and 
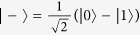
. In this case, it is easy to verify that 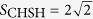.

Traditionally, the PDC source is usually set between Alice and Bob to exclude the locality loophole. Because the lhv theory is refuted, it is not necessary to care about this loophole. Alice can set the PDC source in her laboratory. When the entangled photon pair is generated, she keeps one of them and transfers the other to Bob. As there is no need to care about the locality loophole, the measurement sequences of Alice and Bob do not affect their experimental results. Alice’s measurement can be implemented before that of Bob, and vise versa. If Eve does not intervene, Bell violation must be obtained.

The moment Alice measures on Photon *A*, the state on Photon *B* collapses accordingly. It means that Alice prepares the state on Photon *B* the moment she measures on Photon *A*. Because the photon pair is prepared with the state 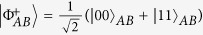, the state on Photon *B* should correlate with what Alice obtains in her measurement. The same correlation can also be obtained with prepare-and-measure procedure: Suppose that single photon is generated from Alice’s source. She randomly chooses the rectilinear basis 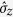 or the diagonal basis 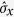, together with random bit value −1 or 1, to prepare Photon *B*. Before Photon *B* is measured, its state correlates with the state chosen by Alice.

If Alice’s basis choices and bit value choices on Photon *B* are totally random, the item 〈*A*_1_*B*_1_〉 in (1) can be calculated as


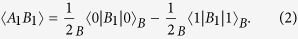


Similarly, one can obtain


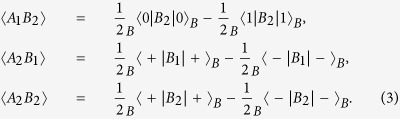


Substituting *B*_1_ and *B*_2_ with 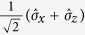 and 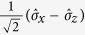, one can obtain that 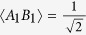, 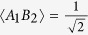, 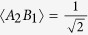, and 
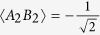
. The value of the CHSH polynomial is calculated to be 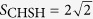. It is the same as that when the entangled state 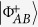 is used. This is because the correlations between the state on Photon *B* and that of Alice are the same in these two cases.

## BB84 protocol with the prepare-and-measure Bell test

The BB84 protocol is a prepare-and-measure QKD protocol. Alice prepares the state on Photon *B* and transfers it to Bob. Bob measures it in randomly chosen basis to read its state. The states |0〉_*B*_ and |+〉_*B*_ are used to encode the bit value 0, while the states |1〉_*B*_ and |−〉_*B*_ are used to encode the bit value 1 (Here the definitions of the bit values in the quantum key distribution and those of the Bell test are different).

The prepare-and-measure BB84 protocol is characterized as follows:

(a) *N* single photons are generated in Alice’s laboratory. She randomly chooses between the diagonal basis 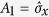 and rectilinear basis 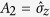 and the random bit values 0 and 1 to prepare her state on the photon. Then the photon is transferred to Bob.

(b) Bob has two modes: with probability *p* he chooses the signal mode and with probability 1 − *p* he chooses the test mode (Only Bob himself is aware of the value of *p*). In the signal mode, Bob’s measurement bases are randomly chosen from 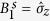 and 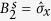. In the test mode, his measurement bases are randomly chosen between 
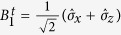
, and 
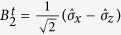
.

(b′) When Bob chooses the signal mode, Alice and Bob publish their measurement bases through the public channel. They keep their measurement outcomes in the same bases as the sifted key bits.

(b″) When Bob chooses the test mode, Alice and Bob announce their basis choices and measurement outcomes through the public channel to calculate the value of *S*_CHSH_.

(c) After the key distribution, Alice and Bob implement error correction (EC) and privacy amplification (PA) on their sift key bits. If secure key bits can be generated, their key distribution task is fulfilled. Or else, their task is failed.

Because Alice randomly chooses her basis and bit values on Photon *B*, the state on it is 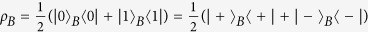 for any third party. The state is uniformly prepared in the conjugated bases. Eve cannot differentiate which state is prepared. If she carries out state distinguishing task on the photon, disturbance must be introduced^43^,^44^. Without loss of any generality, one can assume that Eve interacts on Photon *B* with a probe. If the interaction between the probe and Photon *B* can be characterized as a unitary process, one can obtain


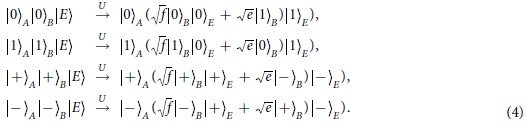


Here |*E*〉 is the blank state on Eve’s probe. *f* and *e* correspond to the probability that the state on Photon *B* is intact and the probability that the state on Photon *B* is changed, respectively. It means that Eve’s intervention introduces quantum bit error rate (QBER) with probability *e*. The amount of information for Alice and Bob used to correct the errors on their bit string is *h*(*e*), with *h*(*x*) = −*x*log_2_*x* − (1 − *x*)log_2_*x* the binary entropy. In the prepare-and-measure QKD, Eve’s ability to attack on the communication can be bounded with the collective attack^3^,^45^. In this case, the rate for Eve to tag Bob’s key bits is upper bounded with^46^,^47^


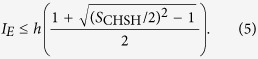


After Eve’s intervention, 〈*A*_1_*B*_1_〉 is recalculated to be


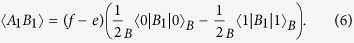


Similarly, one has


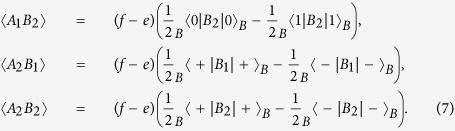


It means Alice’s and Bob’s states are correlated with probability *f*, and anti-correlated with probability *e* that is also known as the QBER. After Eve’s intervention, 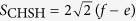 is obtained.

## Refute the detector side channel attack

In the BB84 protocol, Eve’s illegal information is also bounded as^3^,^4^,^7^


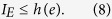


Here *e* is the phase error rate gained on Bob’s state. Strictly speaking, phase error rate is the concept of entangled states. Because of the symmetry of BB84 protocol, however, the phase error rate is estimated from the bit error rate on the results generated from the conjugated bases^4^,^7^. Within the process of Eve’s attack, if she can hide the bit error rates of both bases, Alice and Bob cannot find her existence. In the detector side channel attack, for instance, it is possible for Eve to intervene without introducing any bit error rate.

If Bob’s detectors are imperfect, the detector side channel information leaking problem may exist. Eve can exploit the flaws of Bob’s detectors to carry out the detector side channel attacks.

**Theorem 1** *The detector side channel attacks can be successfully carried out if and only if Eve’s information of Alice’s bit value conditioned on Bob’s measurement outcome is partially or totally certain*. Proof: In the BB84 protocol, Eve interacts with Photon *B* to extract Alice’s state on it. After Bob measuring on the incoming photons, he and Alice declare their basis choices. When considering the individual attack, Eve’s information gain from Alice is bounded by her entropy decrease on her state^3^


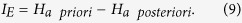


In the detector side channel attack, though drawbacks exist on Bob’s detectors, it is indispensable to assume that no unwanted information can leak out of his laboratory. Or else, the security of QKD cannot be ensured. It means that Eve should control the information leaking from the detectors indirectly. If Alice’s state on Photon *B* is uniformly prepared, *H*_*a priori*_ = 1 is obtained.

Consider that *H*_*a posteriori*_ = ∑_*r*_*P*(*r*)*H*(*i*|*r*), with *P*(*r*) the probability Eve obtains the measurement result *r*, and *H*(*i*|*r*) the information gain of *i* conditioned on *r*. If Eve does not interact with Photon *B*, *H*_*a posteriori*_ = 1 after Bob declaring his basis choices. Thus *I*_*E*_ = 0 and Eve cannot obtain any illegal information on Alice’s state. In the detector side channel attack, *r* has two possible values: the registered and the unregistered, and ∑_*r*_*P*(*r*) = 1 is satisfied. Whether for the registered pulses or for the unregistered pulses, Bob’s measurement outcomes are controlled to be bit value biased. Bob announces Alice the values of *r* after his measurements. The bias of Bob’s measurement outcomes is known to Eve in the detector side channel attack and *H*(*i*|*r*) should be less than 1. Correspondingly, *H*_*a posteriori*_ < 1 and *I*_*E*_ > 0 are satisfied. In some detector side channel attacks, Eve’s uncertainty on Alice’s states is eliminated with the intercept-resend attack. However, only the pulses encoded with Eve’s expected bit values and expected bases are forced to be detected by Bob. Or else, Alice and Bob can find Eve’s presentence according to the correlations between them. In any case, Bob’s measurement outcomes are partially or totally certain to Eve. Thus we end the proof.

In order to refute the detector side channel attack, Alice and Bob should estimate Eve’s information gain from the attack. In practical QKD, time-windows is set for the detectors so that their dark count rate can be decreased. Thus their detection efficiencies are time-dependent. If the time windows of the two detectors are not the same, there are detection mismatches between them. This can be exploited by Eve to launch the so-called time-shift attack. Furthermore, the detector flaws may also be used by Eve to control the detectors to detect unwanted signals. Taking the blinding attack for instance, the detectors can be blinded with strong illuminations so that they are insensitive to single-photon pulses but to strong pulses. Both in the time-shift attack and in the blinding attack, Eve’s *a posteriori* information on Alice’s bit value is partially or even totally deterministic.

In practical Bell test with EPR pairs, the quantum channel is lossy and one has to consider the detection loophole. It means that any pulse in quantum channel cannot represent the others in violating the CHSH inequality. If the devices are inefficient, fair-sampling assumption must be made to obtain the Bell violation. If Alice and Bob can implement quantum non-demolition measurement on the photon number, they differentiate the vacuum pulses from the non-vacuum pulses. With this technique, they can remove the channel loss. Now that the lhv theory has be excluded by recent experiments, it is reasonable to assume that the movements all pulses obey the quantum principles and all photon pulses experience the same transmission situation. Thus one can sample the behaviors of some of the incoming pulses on the behalf of those of the others.

We consider the QKD with active basis choice. Bob has two detectors *D*_0_ and *D*_1_ to decode the key bits 0 and 1 encoded on Alice’s pulses. Their detection efficiencies are assumed to be *η*_0_ and *η*_1_, respectively. *D*_0_ and *D*_1_ are manufactured to be the same so that *η*_0_ = *η*_1_ = *η* is satisfied when there is no detector side channel attack. We assume that Bob has another detector *D*_*t*_ whose detection efficiency is *η*_*t*_. *D*_*t*_ is also the same as *D*_0_ and *D*_1_ apart from its big time window. The time window of *D*_*t*_ is big enough so that its detection efficiency is stably kept within the whole time windows of both *D*_0_ and *D*_1_. This can be realized by keeping *D*_*t*_ switched on within this period of time. When Bob receives the pulses from Alice, she randomly detect them directly with *D*_*t*_ or decode their key bits with *D*_0_ and *D*_1_ in randomly chosen bases. When detector side channel attacks randomly happen on *D*_0_ and *D*_1_, *η*_0_ and *η*_1_ decrease. For Bob, the detection efficiency he can observe for *D*_0_ and *D*_1_ is 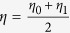. According to Eqs (2) and (3), the value of the CHSH polynomial should be normalized as


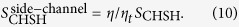


This relation can also be explained with the theory raised by Garg and Mermin where the photons that can be detected by *D*_*t*_ but are missed by *D*_0_ and *D*_1_ can only be assigned with the bit value 0^48^. Accordingly, the illegal information gain Eve obtained within her detector side channel attack is calculated to be


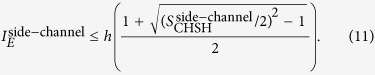


## The performance of the present protocol

In practical QKD, instead of single-photon source, weaken coherent sources are used for Alice to encode her key bits. Compared with single-photon source, multi-photon pulses exist. It is proven that Eve can exploit the multi-photon pulses to launch the so-called PNS attack. Thus decoy states are usually added in practical QKD protocol to beat this attack^12^,^13^,^14^. We consider the practical decoy state protocol raised by Ma *et al*. where a signal source, a weaker decoy source and a vacuum decoy source are used^14^. The intensities of the signal source and the weaker decoy source are *μ* and *ν*, respectively. After randomization of the phase, the photon number distributions of the sources obey


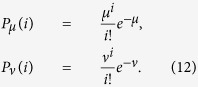


Here *i* is the photon number in the pulses.

Alice randomly chooses the signal source and the decoy sources to encode her bit values and transfers them to Bob. When Bob takes the pulses from Alice, with probability *p* he chooses the signal mode that he measures the incoming pulses randomly in 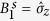 and 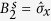 to extract the key bits on them. With probability *p*^′^, he chooses the test mode that his measurement bases are randomly chosen between 
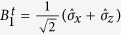
, and 
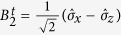
. With probability 1 − *p* − *p*′, however, Bob directly measures Alice’s pulses in his detector *D*_*t*_. The gains on the detectors *D*_0_/*D*_1_, and *D*_*t*_ write


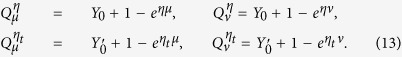


Here *Y*_0_ and 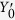 are the dark counts on *D*_0_/*D*_1_ and *D*_*t*_ that can be estimated from the vacuum decoy state. After Bob’s measurements, Alice announces Bob her state choices with which Bob can calculate the values of *η* and *η*_*t*_.

After the EC and PA, the final key generation rate is


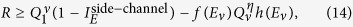


where 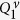 is the gain on the untagged pulses of the weaker decoy source, *f*(*E*_*ν*_) is error correction efficiency, and *E*_*ν*_ is the total QBER on the sifted key bits generated from the decoy source. Here we choose the weaker decoy source for key generation because the multi-photon pulses affect the value of the CHSH polynomial greatly. Numerical results shows that no Bell violation can be obtained when the intensity of the signal source is greater than 0.659 even if *D*_0_ and *D*_1_ have perfect detection efficiency. The fraction of the multi-photon pulses in the decoy source is comparably small, however, thus we assume Alice and Bob use it to generate the key bits.

We will give some numerical simulations on the performance of the QKD with prepare-and-measure Bell test. We will use the setup parameters from the QKD experiment completed by Gobby, Yuan and Shields (GYS)^49^ that has also been taken used for numerical simulation in ref. 14. Namely, the transferring coefficient is *β* = 0.21d*B*/*km*, the detector’s detection efficiency is *η*_*B*_ = 4.5%, the misalignment coefficient is *e*_*d*_ = 3.3%, and the dark count rate *Y*_0_ = 1.7 × 10^−6^. The intensities of the signal pulses, weaker decoy pulses and vacuum pulses are 0.48, 0.1 and 0, respectively. One thing should be pointed out that we will not consider the contribution of the misalignment to the value of the CHSH polynomial.

Firstly, we want to show the performances of the present protocol under the blinding attack and the time-shift attack. For blinding attack, Eve controls the basis of Bob. When Bob’s basis choices coincide with hers, there are efficient registers on his measurement settings. Or else, no clicking event happens. Thus one can obtain *η*_*t*_ = 2*η*, and the value of CHSH polynomial is 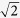 for perfect single-photon source. It means that no secure key bits can be generated between Alice and Bob. For the time-shift attack, the CHSH inequality after the time-shift attack is calculated to be 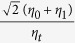. In this attack, however, Eve’s illegal information gain is calculated to be 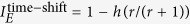, with 
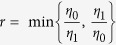
. For simplicity of discussion, one can assume that *η*_0_ < *η*_1_. It is apparent that the big the value 
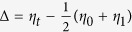
 is, the more key bits should be sacrificed for PA. The amount of information Alice and Bob should sacrifice for PA is 
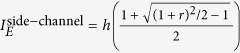
.

**Figure 1:**
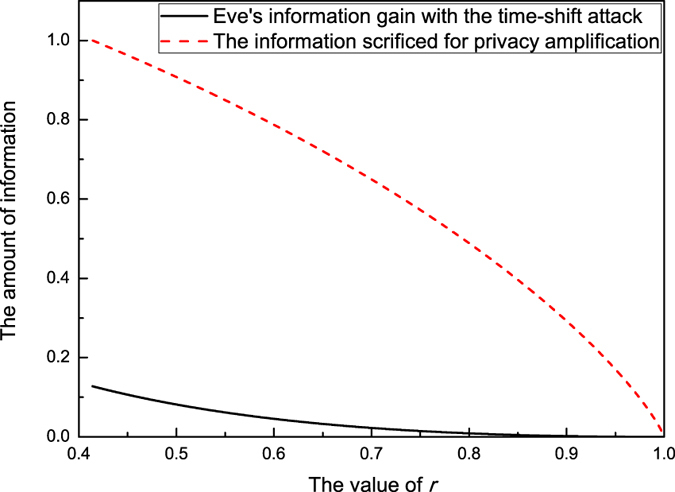
The solid line represents Eve’s information gain within her time-shift attack. The dash line represents the amount of information Alice and Bob should sacrifice for PA after Eve’s time-shift attack.

**Figure 2:**
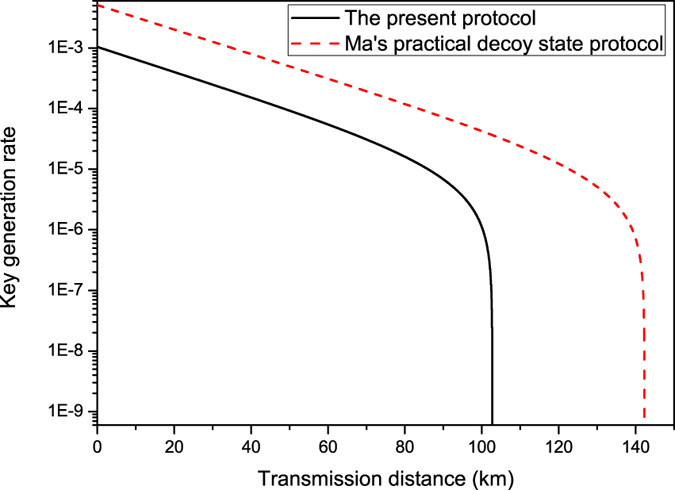
The solid line represents the relation between the key generation rate and the transmission distance of the present protocol. The dash line characterize the relation between the key generation rate and the transmission distance in ref. 14.

From Fig. 1, it is apparent that the amount of information (dash line) Alice and Bob sacrificed for PA is greater than that (solid line) Eve obtained in her time-shift attack. Thus the present protocol is secure under the time-shift attack. The value of *r* begins from 0.414 because 
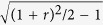
 required that 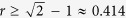. When 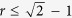, however, one can obtain *S*_CHSH_ ≤ 2. It means that the classical bound of the CHSH inequality cannot be violated and no secure key bit can be generated. We can also compare the key generation rate of the present protocol with that of the practical decoy state QKD^14^. In Fig. 2, one can see that the key generation rate of the present protocol is small than that in ref. 14. This is because we generate the key bits from the weaker decoy state whose intensity is smaller than that of the signal state. Furthermore, the transmission distance of the present protocol can reach about 103 km (solid line). This distance is also shorter than that in ref. 14 (dash line).

## Discussion and Conclusion

In this paper, the QKD protocol with prepare-and-measure Bell test has been proposed. Though the security of the present protocol is based on Bell’s theorem, it is different with the DI-QKD protocol^46^,^47^ and the Ekert91 protocol^2^. First, there is no need to care about the detection efficiency. In the present protocol, only the registered photons are used to calculate the CHSH polynomial. Second, entanglement is not required in the present protocol. The protocol is implemented in a prepare-and-measure way. Furthermore, the Bell test in the present protocol is only carried out at Bob’s side. Our protocol is also different with the detection device-independent QKD protocols of the ref. 50. In the detection device-independent protocols, Alice and Bob have characterized sources but uncharacterized detectors. In the present protocol, however, Bob’s detectors are partially characterized. We assume that the detectors have the same attributions. Furthermore, Bob can control the time window of the testing detector.

## Additional Information

**How to cite this article**: Tan, Y.-g. Quantum key distribution with prepare-and-measure Bell test. *Sci. Rep*. **6**, 35032; doi: 10.1038/srep35032 (2016).
